# Identification of *O*-Linked Glycoproteins Binding to the Lectin *Helix pomatia* Agglutinin as Markers of Metastatic Colorectal Cancer

**DOI:** 10.1371/journal.pone.0138345

**Published:** 2015-10-23

**Authors:** Diluka Peiris, Marlène Ossondo, Simon Fry, Marilena Loizidou, Juliette Smith-Ravin, Miriam V. Dwek

**Affiliations:** 1 Department of Biomedical Sciences, Faculty of Science and Technology, University of Westminster, London, United Kingdom; 2 Universite des Antilles et de la Guyane, Département Scientifique Interfacultaire, EA929 AIHP-GEODE (BIOSPHERES), Campus de Schœlcher, Martinique; 3 Division of Surgery and Interventional Science, University College London School of Life and Medical Sciences, Royal Free Campus, Pond Street, London, United Kingdom; University of Liverpool, UNITED KINGDOM

## Abstract

**Background:**

Protein glycosylation is an important post-translational modification shown to be altered in all tumour types studied to date. Mucin glycoproteins have been established as important carriers of *O-*linked glycans but other glycoproteins exhibiting altered glycosylation repertoires have yet to be identified but offer potential as biomarkers for metastatic cancer.

**Methodology:**

In this study a glycoproteomic approach was used to identify glycoproteins exhibiting alterations in glycosylation in colorectal cancer and to evaluate the changes in *O-*linked glycosylation in the context of the p53 and KRAS (codon 12/13) mutation status. Affinity purification with the carbohydrate binding protein from *Helix pomatia* agglutinin (HPA) was coupled to 2-dimensional gel electrophoresis with mass spectrometry to enable the identification of low abundance *O-*linked glycoproteins from human colorectal cancer specimens.

**Results:**

Aberrant *O-*linked glycosylation was observed to be an early event that occurred irrespective of the p53 and KRAS status and correlating with metastatic colorectal cancer. Affinity purification using the lectin HPA followed by proteomic analysis revealed annexin 4, annexin 5 and CLCA1 to be increased in the metastatic colorectal cancer specimens. The results were validated using a further independent set of specimens and this showed a significant association between the staining score for annexin 4 and HPA and the time to metastasis; independently (annexin A4: Chi square 11.45, P = 0.0007; HPA: Chi square 9.065, P = 0.0026) and in combination (annexin 4 and HPA combined: Chi square 13.47; P = 0.0002).

**Conclusion:**

Glycoproteins showing changes in *O-*linked glycosylation in metastatic colorectal cancer have been identified. The glycosylation changes were independent of p53 and KRAS status. These proteins offer potential for further exploration as biomarkers and potential targets for metastatic colorectal cancer.

## Background

Glycosylation is an important post translational modification that has been shown to be altered in all cancer types studied to date. The genes coding for enzymes involved in glycosylation reactions account for approximately 1% of the human genome explaining the myriad of glycosylation changes reported in cancer. Metastasis, the dissemination of cells from the primary tumour to distant organs, is a major problem affecting patients diagnosed with colorectal cancer (CRC) [1]. Accordingly, early diagnosis of metastatic CRC is an important aim and new prognostic markers are needed to identify patients at the highest risk of developing metastases to enable oncologists to tailor treatment strategies to high risk groups of patients.Alterations in glycosylation have been shown to be associated with the metastatic behaviour of tumour cells [2,3] and may be identified using carbohydrate binding proteins, lectins, isolated from a wide variety of organisms including bacteria, plants, invertebrates and vertebrates [4]. The lectin *Helix pomatia* agglutinin (HPA) recognises *O-*linked glycan structures [5,6] and was first shown to bind preferentially to breast cancer from patients with poor prognosis [2] [7]. The utility of HPA for detecting metastatic cancer has since been shown for a range of solid tumours including those of the gastrointestinal tract, for example, colorectal, gastric and oesophageal tumours [8–11]. The glycoproteins recognized by HPA have been investigated in CRC derived cell lines and this revealed that the utility of HPA lies in its ability to simultaneously bind to many glycoproteins involved in a range of pro-metastatic functions including cell migration, anti-apoptosis and cell signalling [12]. HPA has been shown to bind the same glycoproteins in breast cancer as those detected in HT29 cells suggestive that HPA recognises changes in glycosylation that are common across solid tumour types. HPA recognises glycoproteins modified by the attachment of *O-*linked *N*-acetylgalactosamine (*O-*GalNAc) and *N*-acetylglucosamine (*O-*GlcNAc) [13]. Cancer associated changes in *O-*linked glycosylation have been recognised for some time but there have been relatively few studies concerned with mapping the proteins to which the altered glycans are attached.In the current study, the lectin HPA was used to identify *O-*linked glycoproteins of CRC tissues that were metastatic to the lymph nodes. Proteins were pre-enriched by lectin affinity chromatography and separated by two–dimensional gel electrophoresis (2-DE) followed by identification using mass spectrometry (MS). Verification of the glycoproteins identified was performed by immunohistochemistry and Western blotting. The correlation between HPA binding; mutated P53 in KRAS and the CEA status of the tissue samples was evaluated with the aim of understanding *O-*linked glycosylation in the context of other markers for CRC. A validation study with a further collection of CRC specimens independent of the initial sample cohort showed that the changes in *O-*linked glycosylation recognised by HPA remain associated with poor prognosis CRC.

## Materials and Methods

Chemicals were obtained from Sigma-Aldrich, Poole, Dorset, UK, unless otherwise stated.

### Patient samples

The proteomic study included 27 patients with primary CRC, known to be free of liver metastases, all of whom underwent surgical resection at the University Hospital, Martinique (S1 Table). The study was approved by the Ethics Committee of the Medical School of Martinique and the University Research Ethics Committee (UREC) of the University of Westminster. All patients gave written informed consent for their samples to be used in research. Samples were snap-frozen after surgery and equivalent samples were fixed in formaldehyde and embedded in paraffin for histopathological studies. For each cancer tissue sample, normal tissue was collected from tumour free areas in the neighbouring mucosa within a radius of 4 cm of the tumour. Patients were categorised into two groups based on the degree of the involvement of the regional lymph nodes. All the tissues used in the investigation were examined and verified for the presence of tumour cells using haematoxylin and eosin-stained slides. The validation work was performed using paraffin-was embedded blocks of CRC tissue collected at University College London Hospitals (prior to 2006) and used in accordance with the Human Tissue Act (2008).

### Histochemistry

The immunohistochemistry used the horseradish-peroxidase (HRP) detection system (EnVision kit plus Dual link system-HRP complex, Anti-Mouse/Rabbit-HRP, Dako, Carpinteria, CA, USA) according to the manufacturer's instructions. Briefly, 4 μm thick sections from the formalin-fixed paraffin-wax embedded (FFPE) archival colon tissues (tumours and normal counterparts) were de-paraffinized in xylene and rehydrated through a series of graded alcohols (70% - 100%). Antigen retrieval was performed using the Target Retrieval Solution or citrate buffer following manufacturer’s instructions. Endogenous peroxidase activity was quenched by incubation with the Dual Endogenous Enzyme block (Dako, Carpinteria, CA, USA). Sections were incubated with the respective primary antibody: monoclonal anti-p53 (clone DO1; Immunotech, dilution 1:50) or rabbit polyclonal antibody against annexin 4 and 5 (dilution 1:50; Santa Cruz Biotechnology, Santa Cruz, CA, USA). Conditions used for each antibody were as described by the manufacturer. Staining without the primary antibody was performed as a negative control. The Envision system, HRP, anti-mouse or anti-rabbit was used for the detection of the primary antibodies (Dako, Carpinteria, CA, USA) and tissue staining was visualised with diaminobenzidine (DAB) chromogen solution (Dako, Carpinteria, CA, USA). Nuclei were counterstained with haematoxylin for 5 min. The sections were dehydrated through a series of graded alcohols cleared in xylene and mounted in di-n-butyl phthalate in xylene (DPX) mounting medium. Histochemistry to detect HPA binding utilised 10 μg/ml biotinylated lectin in phosphate buffered saline (PBS) with 5% w/v bovine serum albumin (BSA) applied to the slides for 1 h, washed in three changes of PBS with 0.01% v/v Tween-20 and incubated with 5 μg/ml streptavidin-HRP for 30 min. The sections were washed again in changes of PBS with 0.01% v/v Tween-20 and lectin binding was revealed by incubation with 1% w/v DAB and 0.3% v/v H_2_O_2_. The cells were counterstained and mounted as above.

Slides were evaluated under a light microscope and were scored by two independent individuals in a blinded manner. In cases where there was initial disagreement, a consensus was obtained after discussion. P53 was recorded as positive when the tumour cell nuclei were stained, irrespective of the percentage of positive cells. The overall immunoreactive score was determined (for P53) according to the method used previously [6]. IRS is the cumulative value of the intensity of immunostaining (0 (-) for no staining; 1 (+) for weak staining; 2 (++) for moderate staining and 3 (+++) for strong staining) and the percentage of positive tumour cells <5% = 0; 20% = 1; 20–50% = 2; 50–80% = 3 and >80% = 4. For HPA and annexin 4, the same criteria were used. First the percentage of cancer cells staining was assessed and this was then added to the intensity score to give a final score of between 0 and 8. In order to determine the optimum cut-off, the results from all the samples were analysed in terms of their staining score. The score which gave the greatest discrimination between the patients who developed metastatic disease and those who remained disease free (5-year survival data) was used as the cut-off value.

### DNA extraction

Samples of tumour tissue and normal tissue from each patient were used for DNA extraction. Sections of 1 mm^3^ tissue were cut and crushed immediately in 200 μl of lysis buffer pH 7.5 containing 10 mM Tris, 1 mM EDTA, 0.5% v/v SDS, 100 μg/ml proteinase K. Phenol extractions were performed and the DNA was precipitated with ethanol at -20°C. The resulting precipitate was dried at room temperature and re-suspended in sterile Tris-EDTA (TE) buffer at a concentration of approximately 1 mg/ml. DNA concentration was determined using a GeneQuant Pro spectrophotometer (GE Healthcare, Bucks, UK). The samples were stored at +4°C until use.

### DNA amplification

The amplification reaction of the DNA extracted from normal and tumour tissue were performed using 1 μl DNA sample in a total reaction volume of 50 μl containing Promega Master Mix (Ref. M7502) and primers generated by Dr. E. Jullian of Cochin Hospital. The amplification reaction was carried out in two stages, with 12 pM of each primer. The first PCR reaction (KRAS1) was performed with the following primers: KRN5 '(5'-TAAGGCCTGCTGAAAATGAC—3') and KRN3 '(5'-TGAAAATGGTCAGAGAAACC—3') with the following amplification cycle: 95°C for 10 min, 55°C for 1 min, 72°C for 1 min, then 43 cycles of 95°C for 30 sec, 55°C for 30 sec, 72°C for 1 minute, and a final extension at 95°C for 30 sec, 55°C for 30 sec, 72°C for 10 min. The observation of a weak band of low intensity or of the lack of band, after 1.5% w/v agarose gel electrophoresis led to a second amplification reaction. This second PCR reaction (KRAS2) was carried out using 2 to 10 μl of the first reaction KRAS1 and the following primers: KRS5 '(5'—AACTTGTGGTAGTTGGAG -3 ') and KRS3' (5 '- GTTGGATCATATTCGTCC -3') with the following amplification cycle: 95°C for 12 min, 52°C for 1 min, 72°C for 1 min, then 43 cycles of 95°C for 30 sec, 52°C for 30 sec,72°C for 1 min and a final cycle at 95°C for 30 sec, 52°C for 30 sec, 72°C for 10 min. The PCR products from the tumour tissue were sequenced for and mutations in codons 12 and 13 of the KRAS gene were identified (Millegen, France).

### Sample preparation for protein analysis

Prior to the selection of samples for proteomic studies, 7 μm frozen sections were cut and the presence of cancer cells was confirmed by staining with haematoxylin and eosin. Samples containing >60% cancer cells and haemolysis free were selected. Four protein pools were prepared: LN positive (n = 6) CRC (LN+), adjacent healthy tissue (AdLN+); LN negative (n = 5) CRC (LN-) and corresponding healthy tissues (AdLN-). The clinical pathological features of the samples are shown in S1 Table. Proteins were extracted from each sample by washing 50 mg of tissue three times with chilled PBS, homogenising in lectin buffer: 0.05 M TBS, 1 mM CaCl_2_, 1 mM MgCl_2_, pH 7.6 using a hand held homogeniser for 5 min with intermittent cooling on ice. The homogenate was centrifuged at 15,000 x *g* to collect the supernatant. Protein quantification was carried out using the Qubit system (Qiagen, U.K.). The protein yields ranged from 5% to 7% of the wet weight of the tissue. Each of the pools comprised 100 μg of each of the cancer / normal protein preparations of the appropriate samples.

### SDS-PAGE and Western blotting

Proteins were separated by 1-DE sodium dodecyl sulphate polyacrylamide gel electrophoresis (SDS-PAGE) using a 12% gel at 120 V for 1.5 h [14] and stained with Colloidal Coomassie Blue G-250 [15]. Samples separated by SDS-PAGE were transferred to nitrocellulose using a wet blotting system for 1.5 h at 110 V in transfer buffer: 25 mM Tris, 192 mM glycine, 20% v/v methanol, blocked with 2% w/v BSA in TBS-Tween 0.05% v/v overnight and incubated with 5 μg/ml biotinylated HPA for 2 h followed by 1 h incubation with 2 μg/ml streptavidin-HRP and probed with the DAB/H_2_O_2_. For analysis of the CEA levels a murine anti-CEA monoclonal antibody (sc-55547, Santa- Santa-Cruz, CA, USA) was used at 5 μg/ml anti-CEA antibody for 2 h, incubated with 2 μg/ml peroxidase-conjugated anti-mouse IgG for 1 h and visualised as above. To verify the levels of annexin 4 and 5 protein samples were separated and blotted as before and probed with murine monoclonal anti-annexin 4/5 antibodies (sc-46693/ sc-74438, Santa-Cruz, CA, USA) at 5 μg/ml overnight followed by incubation with goat anti-mouse IgG (1 μg/ml) and developed with the enhanced chemiluminescence (ECL) substrate. The signal was captured onto X-ray film using an exposure time of 1 min.

### Enrichment of HPA binding glycoproteins

HPA affinity chromatography was used to enrich and purify HPA binding glycoproteins from the tissue extracts. This allowed qualitative alterations in glycosylation and quantitative changes in protein levels to be assessed. In addition, this step removed high abundance proteins. A 5 ml HPA affinity chromatography column was prepared by coupling 10 mg of HPA to a HiTrap NHS-activated Sepharose column (GE Healthcare, Bucks, UK) according to the manufacturer’s instructions. 2 mg of pooled protein sample was loaded onto the column and eluted with 0.25 M GlcNAc freshly prepared in lectin buffer. Samples were dialysed overnight against TBS and freeze dried. Lyophilised samples were reconstituted in 100 μl urea buffer: 7 M urea, 2 M thiourea, 2% v/v ampholytes 4–7 (GE Healthcare, Bucks, UK), 4% w/v CHAPS and 1% w/v DTT. Protein quantification was carried out as before. The yield of affinity purified protein ranged from 1% to 2% of the total protein pool.

### Two dimensional electrophoresis (2-DE)

Affinity purified glycoproteins were separated by 2-DE. 50 μg of each pooled protein sample was mixed with rehydration buffer: 6 M urea, 2M thiourea, 0.5% v/v CHAPS, 0.4% v/v DTT, 0.5% v/v ampholytes pH 4–7, to give a final volume of 120 μl. The mixture was loaded onto an 11 cm Immobiline pH gradient strip, pH 4-7L (GE Healthcare, Bucks, UK) according to the method described by [16]. Isoelectric focusing was performed using a Multiphor II (GE Healthcare, Bucks, UK) 10,000 Vh (GE Healthcare, 2004). After focusing, the IPG strip was stored at -80°C until separation by SDS-PAGE as described above. Three replicates of each of the affinity purified glycoprotein pools were separated by 2-DE and visualised by staining with Colloidal Coomassie Blue G-250 [15]. Gel images were captured using a GS-800 Densitometer (BioRad, UK).

### Analysis of 2-DE separations

The 2-DE separations were analysed to identify glycoproteins present in different levels in the CRC specimens of patients with LN positive disease. Scanned image files were analysed using the Progenesis PG240 SameSpots software (Nonlinear Dynamics, U.K.), relative protein quantity in a given spot is inferred from the pixel intensity value and converted to a percentage of the total intensity of all protein spots on the gel. Glycoproteins showing altered levels were ranked according to the p-value (one way ANOVA) and the fold-change values.

### Matrix assisted laser desorption ionisation mass spectrometry (MS) analysis

Protein identification was carried out by commercial arrangement with Dr Kevin Bailey, School of Biomedical Sciences, University of Nottingham. Protein spots were excised from the 2-DE separated gels; desalted using reversed phase C18ZipTips (Millipore, UK). 2 μl of desalted peptide sample was spotted onto a single target well on a stainless steel matrix-assisted laser desorption ionisation (MALDI) plate with 1 μl alpha cyano-4-hydroxycinnaminic acid containing internal standard (adeno corticotrophic hormone, ACTH). The target was allowed to air dry and analysed using in a MALDI-MS (MicroMass M@LDI, Waters Corp) operating resolution >10,000 full width at half maximum) in reflection mode. Spectra were acquired at 5 Hz using a nitrogen laser (λ 337 nm) with 10 shots summed per spectra. Data was acquired by randomly sampling from the target plate and the peptide peak list files were generated both manually and automatically. The backgrounds peaks from trypsin and keratin were excluded and the peak lists were loaded into the MASCOT PMF (http://www.matrixscience.com/) database search engine with parameters set as follows: peptide tolerance 0.2 Da; modifications during the digestion process such as oxidized methionine and CAM were accounted for and missed cleavages was set to 1.

### Prediction of post-translational modifications (PTM)

Bioinformatic analysis was undertaken using the NetNGlyc; NetOGlyc; YinOYang; NetPhos tools at http://www.cbs.dtu.dk/services to identify potential N-linked, O-GalNAc, O-GlcNAc and O-phosphate modification sites on the HPA binding proteins identified above.

### Statistical analysis

Comparisons between the 2 groups (lymph node positive/negative CRC) were made using the Student’s independent samples t-test for continuous data and ANOVA for categorical variables. The Pearson coefficient was also used to determine if the levels of HPA or annexin binding were associated with disease-free interval. Survival curves were plotted according to the Kaplan-Meier method and compared using the log-rank test; for all the tests undertaken, P values of <0.05 were taken as statistically significant. All statistical analyses were performed using SPSS version 19 (SPSS Inc., Chicago, IL, USA, IBM Company).

## Results and Discussion

### Proteomic analysis of *O-*linked glycoproteins in CRC

**Fig 1:**
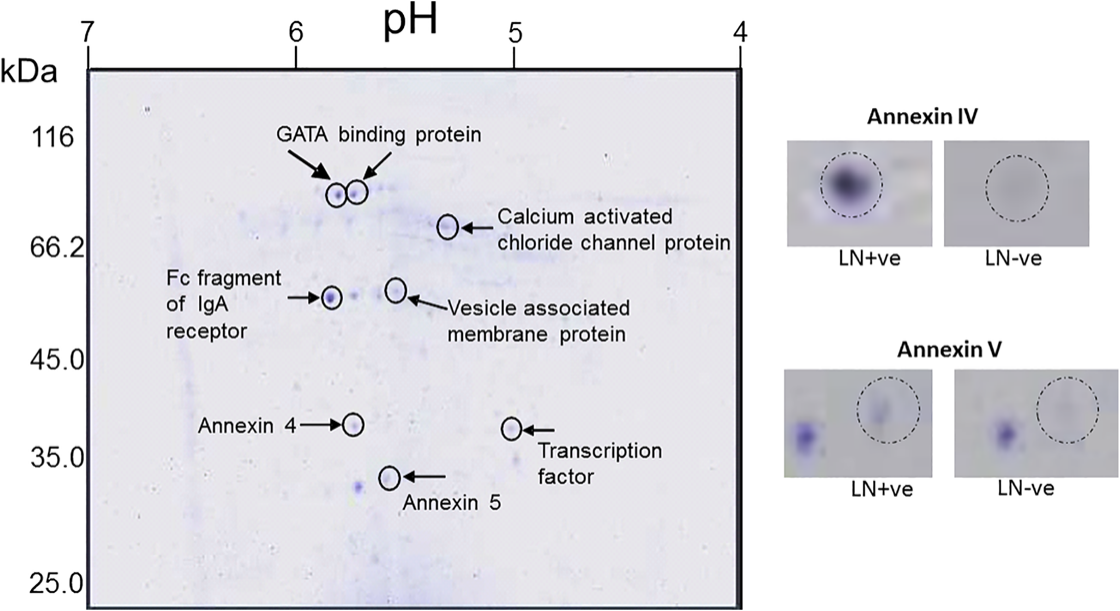
2-DE analysis of *O-*linked glycoproteins affinity purified using HPA. Proteins pooled from LN+ve CRC tissue specimens were affinity purified with HPA and separated by isoelectric focussing on a pH 4–7 IPG strip and then by SDS-PAGE (12%) and visualised using colloidal Coomassie Blue. Inset: annexin 4/5 as indicated in LN+ve compared with LN-ve CRC tissue specimen protein pools. The proteins identified in the MALDI-MS analysis are indicated.

Pooled proteins from CRC tissues were fractionated using HPA affinity chromatography, separated by 1-DE and 2-DE and identified using MS, in a proteomic approach aimed at characterising the *O-*linked glycans elevated in CRC metastatic to the lymph nodes (Fig 1 and S1 Fig). Seven glycoproteins were identified using this approach (Table 1) including the cell membrane proteins annexin 4, annexin 5 and the calcium activated chloride channel protein 1 (CLCA1). Annexin 4 has previously been identified as an HPA binding glycoprotein in the colorectal cancer cell line HT29 [12]. Alongside the increased levels of *O-*linked glycosylation, increased levels of CEA was also identified (S1 Fig) suggest that *N-*linked glycosylation also increase in metastatic CRC cancer.

**Table 1:** Identification of the HPA binding proteins, affinity purified and separated by 2-DE, identified using MALDI-MS. The protein accession number, description, MASCOT score, sequence coverage, nominal mass and predicted pI are indicated. Fold change is the ratio between the mean normalised volume of the spot separated by 2-DE from the LN+ve group and the LN-ve group.

Protein Accession number	Protein description	MASCOT score	Sequence coverage %	Nominal Mass 9kDa)	Predicted *pl*	Fold change
gi|119571135	GATA binding protein	56	23	11953	8.95	-3
gi|31615935	Fc fragment of IgA receptor	59	22	23642	7.12	3.5
gi|223468574	Vesicle associated membrate protein	46	20	20336	9.39	-2.8
gi|119590003	Transcription factor	48	18	37679	5.17	2.5
gi|4585469	Calcium-activated chloride channel protein 1	130	11	10081	5.97	2.5
gi|114577910	Annexin IV	99	25	31535	5.36	3.2
gi|157831404	Annexin V	120	25	35839	4.98	2.8

### *O-*linked glycosylation, p53 and *KRAS* mutation status

**Fig 2:**
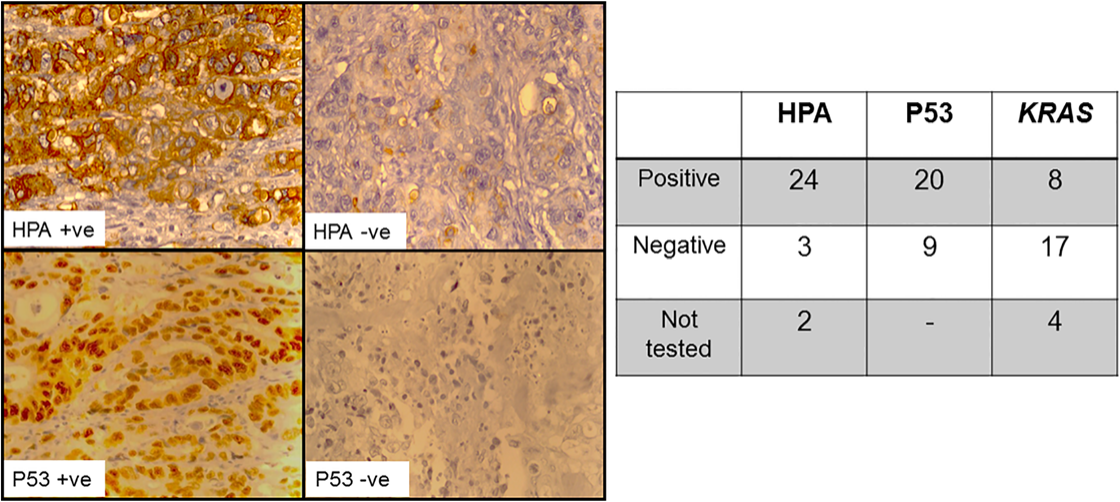
HPA lectin and P53 immunohistochemistry of CRC tissue specimens. Left: light microscopy images of colorectal tissue sections (5 μm) incubated with HPA (10 μg/ml) and streptavidin-HRP (5 μg/ml) or incubated with the anti-P53 antibody (1:200) and biotinylated horse rabbit anti-mouse IgG (1:1000) as indicated. The brown colouration indicates the peroxidase reaction with DAB/H_2_O_2_, the nuclei were counterstained with haematoxylin (blue). Magnification X400. Right: Table showing results for HPA, P53 and *KRAS* analysis.

A histochemical approach was used with HPA as a marker of *O-*linked glycosylation status. The majority of the samples were reactive with the lectin (n = 24, of total n = 27 cases) and exhibited intense cell membrane staining with fewer cases showing granular cytoplasmic staining, perhaps representing the binding of the lectin to glycoconjugates in transit through the Golgi apparatus (Fig 2). The light microscope does not provide sufficient resolution to allow close interrogation of the intracellular organelles of the tissues that bound HPA. It is possible that some of the glycoproteins identified in this study are, therefore, proteins in transit through the secretory pathway, including the Golgi apparatus, however, much of the HPA binding observed using immunohistochemistry was cell membrane associated. There was no relationship between HPA staining intensity and patient age, sex, tumour grade or LN status but this may reflect the relatively few samples in this initial analysis (data not shown). The anti-p53 antibody was strongly reactive in 13 of the 29 cases and weakly reactive in a further 7 cases (Fig 2) and the *O-*linked glycosylation status did not correlate with the P53 staining. All of the P53 positive cases (n = 15) bound HPA as well as some of the P53 negative cases (n = 8). The *KRAS* mutation status was assessed using a PCR based approach and 8 of the 25 evaluable specimens exhibited mutated *KRAS* in either codon 12 or 13 (26% of the cases), this compared favourably with the reported prevalence in CRC of between 30–40% [17]. Of 8 samples with mutated *KRAS*, four were concurrently positive for HPA staining and all of these were P53 positive. Taken together these results are suggestive that *O-*linked glycosylation changes occur early in the aetiology of CRC.

### Validation of the results of the proteomic analysis

**Fig 3:**
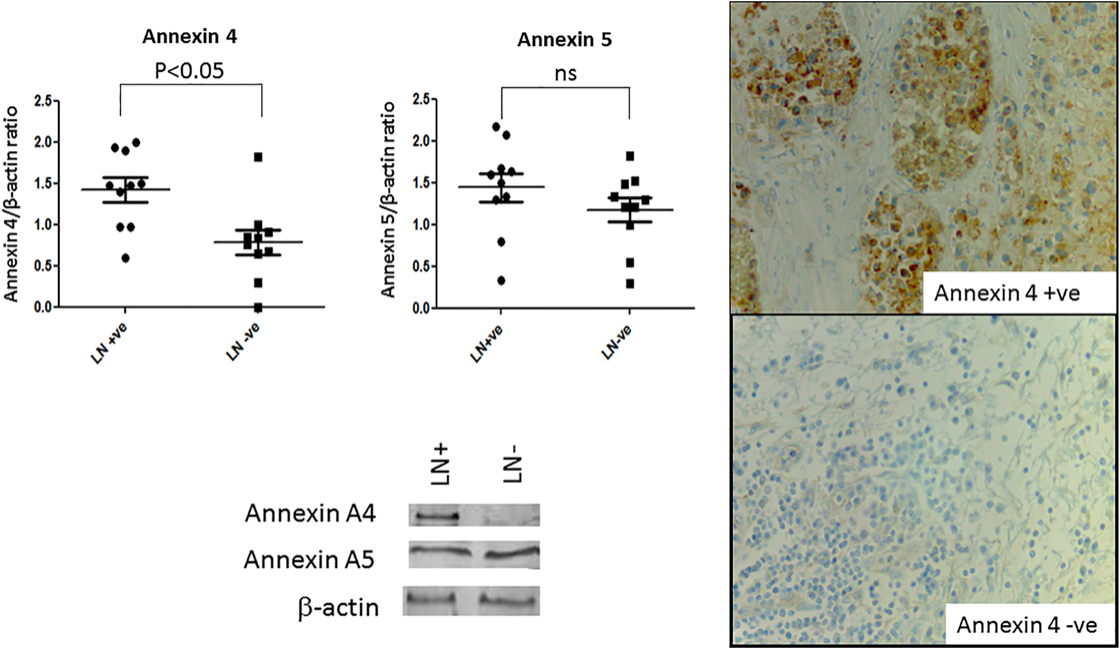
Evaluation of annexin 4 and 5 levels in CRC tissue specimens. Left: 10 μg of pooled proteins were separated by SDS-PAGE using samples from patients with LN positive CRC (LN+ve) and LN negative CRC (LN-ve) blotted to nitrocellulose and probed with either an annexin 4 or 5 antibody followed by incubation with anti-mouse IgG and visualisation using DAB/H_2_O_2_. Upper panel: Annexin 4/5 levels relative to actin levels using densitometric analysis of Western blot data, the P value refers to unpaired Student *t*-test result, NS = not significant. Lower panel: Western blot analysis of annexin 4/5 and corresponding β-actin blot is shown for two individual patient samples, either LN+ve or LN-ve as indicated. Right: 4 μm sections of FFPE CRC probed with anti-annexin 4 antibody. Binding was visualised using DAB/H_2_O_2_ (brown) the nuclei were counterstained with haematoxylin (blue), magnification X400.

**Fig 4:**
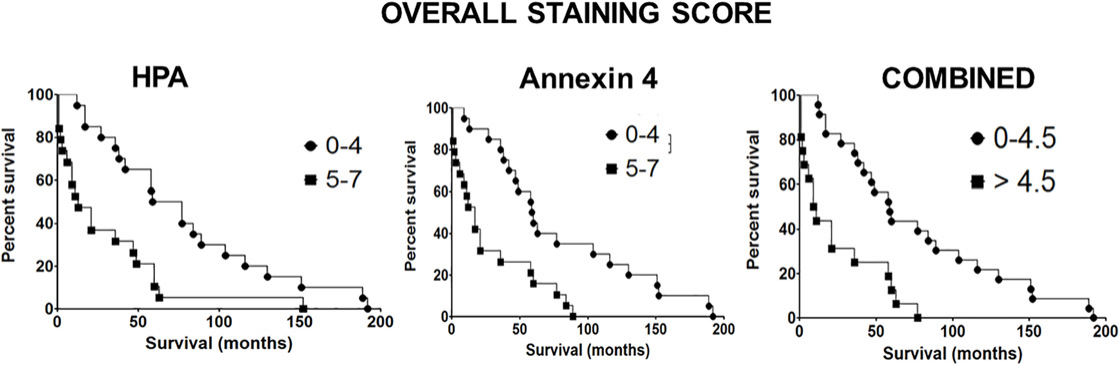
Kaplan Meier survival curves. Upper: The scores for HPA or anti-annexin 4 antibody staining as indicated. Lower: Combined scores for HPA and anti-annexin 4 staining.

Annexin 4 and annexin 5 levels were examined using Western blotting, Fig 3. After normalisation to the β-actin levels, a significant increase in the level of annexin 4 (but not annexin 5) was observed in the CRC tissue specimens that had metastasised to the lymph nodes at the time of diagnosis (student t-test, P = 0.05). Similarly, Western blots were probed with HPA and an increase in the HPA binding glycoproteins was observed in samples from lymph node positive patients (data not shown). This is consistent with findings of other studies reporting an increase in *O-*linked glycosylation in poor prognosis metastatic CRC cancer. To determine whether annexin 4 and HPA binding correlated with poor prognosis CRC an additional series of 35 CRC specimens with patient follow up data were assessed using immunohistochemistry. When the percentage of cells staining (0–100%) and the aggregate staining score (scale 0–7) were considered, both were significantly inversely correlated with patient survival, Fig 4. For example, when the aggregate staining score for HPA was taken as ≥5 the median survival was 13 months compared with 68 months for a cut off ≤5 (Chi square test 9.065; P value = 0.0026); for annexin 4: when a cut off for the aggregate staining score of ≥5 was taken the median survival was 17 months; in contrast a score of ≤5 was associated with a median survival of 59.6 months (Chi square test 11.45; P value = 0.0007). When the results for both annexin 4 and HPA were combined these remained significantly inversely associated with survival after CRC, Fig 4. For annexin 4: taking a cut off of ≥60% of cells staining, the median survival was 11 months compared with 58 months for ≤60% cells staining (Chi square test 7.466; P value = 0.0063);. For HPA: ≥50% cell staining resulted in a median survival of 11.5 months; but when ≤50% cell staining was taken median survival was 60 months (Chi square test 11.74; P value = 0.0006), data not shown. These findings illustrate that the binding of HPA and anti-annexin 4 antibodies to tissue sections of CRC are indicators of poor prognosis. Both the *O-*linked glycosylation–as measured by HPA staining–and the levels of annexin 4 are markers of poor prognosis CRC. The complexity of the proteome renders tumour marker discovery work challenging; nevertheless, there is a need for the identification of biomarkers and new targets for the treatment of CRC. Of the many post translational modifications to occur on proteins, glycosylation is the most abundant and diverse [18] and aberrations in protein glycosylation reported in cancer [19] offer potential for identification of cancer-specific changes. Carbohydrate binding proteins, lectins, have previously been described for the up-front capture of cancer-associated glycoproteins [20, 21]. There has been considerable interest in the glycoproteins recognised by the lectin HPA as the involvement of HPA binding epitopes in the metastatic process has been established using cancer cell lines derived from human tumour tissues [22]. Integrin α5 and α6 and annexin 2 and 4 have previously been identified as the main HPA binding partners in the metastatic CRC cell line HT29 [12]. In this study proteins from CRC tissue samples were pre-fractionated using HPA affinity chromatography thereby allowing low abundance proteins to be isolated and identified. Differences in the HPA binding glycoproteins in specimens that had metastasised to the lymph nodes at the time of diagnosis were apparent when the glycoproteins were separated by 1-DE and blotted to nitrocellulose—indicating an overall increase in *O-*linked glycosylation; likely to be associated with Tn and sialyl-Tn antigen expression in cancer [23]. When HPA was used to probe Western blots of the cancer protein preparations, the number of bands and their intensity varied compared with when the proteins had been pre-fractionated by lectin affinity chromatography (S1 Fig). This may reflect subtle differences in binding when HPA was covalently immobilised to an affinity matrix compared to when the lectin was in free solution. The identification of the HPA binding glycoproteins of CRC was a significant aim of this work, these were compared to the levels of CEA from the same tissue specimens and confirmed to be independent of the CEA levels (S1 Fig). Whilst the study of mucins was beyond the scope of this current work, clearly the levels of MUC1 and MUC2 and their HPA binding properties is a matter of some interest. Similarly, the intracellular localisation of the HPA binding proteins is of interest. It is not technically feasible to fractionate cellular organelles when frozen cancer tissue samples are used as the starting material and so we were unable to determine if glycoproteins from the Golgi apparatus were purified using the affinity chromatography step. The primary aim was to identify proteins that bind the lectin HPA and that are altered in metastatic and non-metastatic CRC. As the tissue-staining using HPA showed both intracellular and intense membrane staining, we hypothesise that the HPA binding glycoproteins identified in this study are likely to be derived from the cell membrane, the cytosol and potentially, from the Golgi apparatus. Proteomic analysis of affinity purified HPA binding proteins revealed seven glycoproteins including annexin 4 and 5 and CLCA1 that were increased in the preparations from the metastatic cancer tissues. Annexin 4 has already been shown in a separate study to be recognised by HPA in the CRC cell line HT29 [12]. All three of these proteins have previously been described in relation to CRC but the proteins have not previously been identified through pre-enrichment via *O-*linked glycan moeities. Annexins are a family of calcium-regulated phospholipid and carbohydrate binding proteins with diverse intra- and extracellular roles in range of cellular processes including signalling, ion transport, cell division and apoptosis [24]. Alterations in the levels of annexins have been associated with tumourigenesis in several types of tumours [25] and increased levels of annexin 4 and 5 have previously been reported in primary CRC compared with the normal colon tissue [12, 26–28]. Annexin 4 was found to be up-regulated in renal carcinoma patient tissue samples compared to adjacent healthy tissues [29]. Several other members of the annexin superfamily, specifically annexin 1, 2, 3 and 7 are present in elevated levels in CRC [, 27,30,31]. Increased levels of annexin 4 have been linked to a loss of cell-to-cell adhesion and increased tumour cell dissemination of the human breast cancer cell line MCF7 [29] supporting the observations here that increased levels of annexin 4 are associated with an aggressive metastatic phenotype. Annexin 5 levels have been reported to be increased in liver metastasis of CRC [32]. In this study annexin 4 was identified through lectin (HPA) affinity chromatography which captured the glycoprotein through its glycan (mainly Tn-antigen) moieties. Therefore, whilst the protein levels of annexin 4 have previously been shown to be altered in CRC, this is, to our knowledge, the first report in which annexin 4 glycosylation has been recognised as being altered in CRC tissue samples and levels correlated with poor prognosis CRC. The importance of cell membrane ion channels for the development of cancer has been well established. CLCA1 and CLCA2 were originally thought to form Ca+2 activated chloride channels, however evidence is accumulating that these are accessory proteins, found in association with Ca^2+^ channels. The role of CLCA1/2 in cancer remains unclear: CLCA1/2 have been reported to be down-regulated in CRC suggesting a tumour suppressive role [33]. In contrast, conjugation of CLCA2 to β4 integrin has been shown to mediate cancer cell metastasis [34]. A hypothesis to explain the increased levels of the CLCA1 protein detected in this study is that it might act as an adhesion partner to β4 integrin in CRC, as β4 integrin has previously been shown to bind the lectin HPA, however, further work is required to explore this idea more fully. Alongside the GalNAc binding properties of HPA [5,6] the lectin also binds *O-*GlcNAc containing glycoproteins [13]. *O-*GlcNAcylation acts as an alternative signalling pathway to phosphorylation [35] and many oncogene and tumour suppressor gene products harbour this post-translational modification [36], for example, *O-*GlcNAcylation of p53 has been shown to regulate the protein activity and stability by blocking ubiquitin-dependent proteolysis [37]. It is notable that a significant increase in the *O-*GlcNAcylation in CRC tissues has been reported when compared to healthy tissues [38]. Bioinformatic analysis was undertaken by inputting the amino acid sequences for of the proteins identified in this study into the YinOYang and NetPhosp tools at www.cbs.dtu.dk. The results indicated that all of the proteins harbour potential *O-*GlcNAcylation and phosphorylation sites (S2 Fig).Elevated levels of CEA have been proposed as a predictor of CRC liver metastasis [39, 40]. In the current study a positive correlation was observed between CEA and annexin A4/A5 levels from the pooled protein samples, however a detailed investigation using individual samples would be needed to explore any associations further. This study has shown that HPA binds to CRC proteins of the same relative molecular mass as CEA but HPA also recognised other lower molecular weight entities described above. Notably, HPA binding epitopes were present in the CRC samples which were negative for mutated KRAS and P53 supporting the hypothesis that changes in *O-*linked glycosylation is an event that occurs early in the aetiology of CRC. Future investigations of microsatellite instability would help to elucidate whether the *O-*linked glycosylation changes observed in CRC arise from chromosomal instability, ineffective DNA mis-match repair mechanisms or potentially a combination of both.

## Conclusion

This study has identified annexin 4 as a major carrier of *O-*linked glycans in lymph node positive CRC tissue samples. The levels and intensity of staining of tissue sections with anti-annexin 4 antibodies and with HPA was associated with significantly poorer survival after CRC. Further work will seek to map the glycans of annexin 4 and to explore the function of this protein in metastatic CRC. In order to reveal useful biomarkers for metastatic CRC a larger, more diverse, CRC patient population will be investigated in future studies.

## Supporting Information

S1 Table Clinico-pathological features of the samples used in the proteomic, initial HPA, P53 and *KRAS* study. (DOCX)

S1 Fig CRC and adjacent normal tissue, assessed using three techniques: 1-DE separation, lectin affinity purification and Western blot analysis. Pooled proteins were prepared from samples of patients with LN positive CRC (LN+ve), adjacent normal tissue (AdLN+ve), LN negative CRC (LN-ve) and adjacent normal tissue (AdLN-ve) and separated using 12% SDS-PAGE. Panel A: proteins, 15 μg/well, separated by SDS-PAGE and stained with colloidal Coomassie blue. Panel B: proteins separated as before, transferred to nitrocellulose and probed with 5 μg/ml biotinylated HPA, 2 μg/ml streptavidin-HRP and visualised using DAB/H_2_O_2_. The arrows indicate proteins that were present in the cancer specimens but not in adjacent normal tissue, these were subsequently identified as GATA binding protein 1 and calcium-activated chloride channel protein 1. Panel C: 10 μg of proteins purified using HPA affinity chromatography, separated by SDS-PAGE and visualised by silver staining. Panel D: Densitometric analysis of Western blots in which samples were separated by SDS-PAGE and transferred to nitrocellulose and probed with anti-CEA antibody.
(TIF)

S2 Fig Predicted glycosylation and phosphorylation sites of the HPA binding glycoproteins identified in the MALDI-MS analysis, the predictions indicate likely *O-*linked GalNAc and *O-*GlcNAc glycosylation sites on all the proteins identified. (DOCX)
