# Supplementary material for: Identification of O-Linked Glycoproteins Binding to the Lectin Helix pomatia Agglutinin as Markers of Metastatic Colorectal Cancer
Source: PLoS One. 2015 Oct 23;10(10):e0138345. doi: 10.1371/journal.pone.0138345 (PMC4619703; doi:10.1371/journal.pone.0138345)
Supplement: S1 Table — (DOCX) [file pone.0138345.s001.docx]

|  | **LN +VE** | **LN-VE** |
| --- | --- | --- |
| n= | 15 | 13 |
| Gender  Male (n=)  Female (n=) | 8  7 | 4  9 |
| Age  (Mean average) | 77 | 65 |
| Grade  T2 (n=)  T3/T4 (n=) | 1  14 | 4  9 |

**S1 Table**
