# Supplementary figures and images for: Identification of O-Linked Glycoproteins Binding to the Lectin Helix pomatia Agglutinin as Markers of Metastatic Colorectal Cancer
Source: PLoS One. 2015 Oct 23;10(10):e0138345. doi: 10.1371/journal.pone.0138345 (PMC4619703; doi:10.1371/journal.pone.0138345)

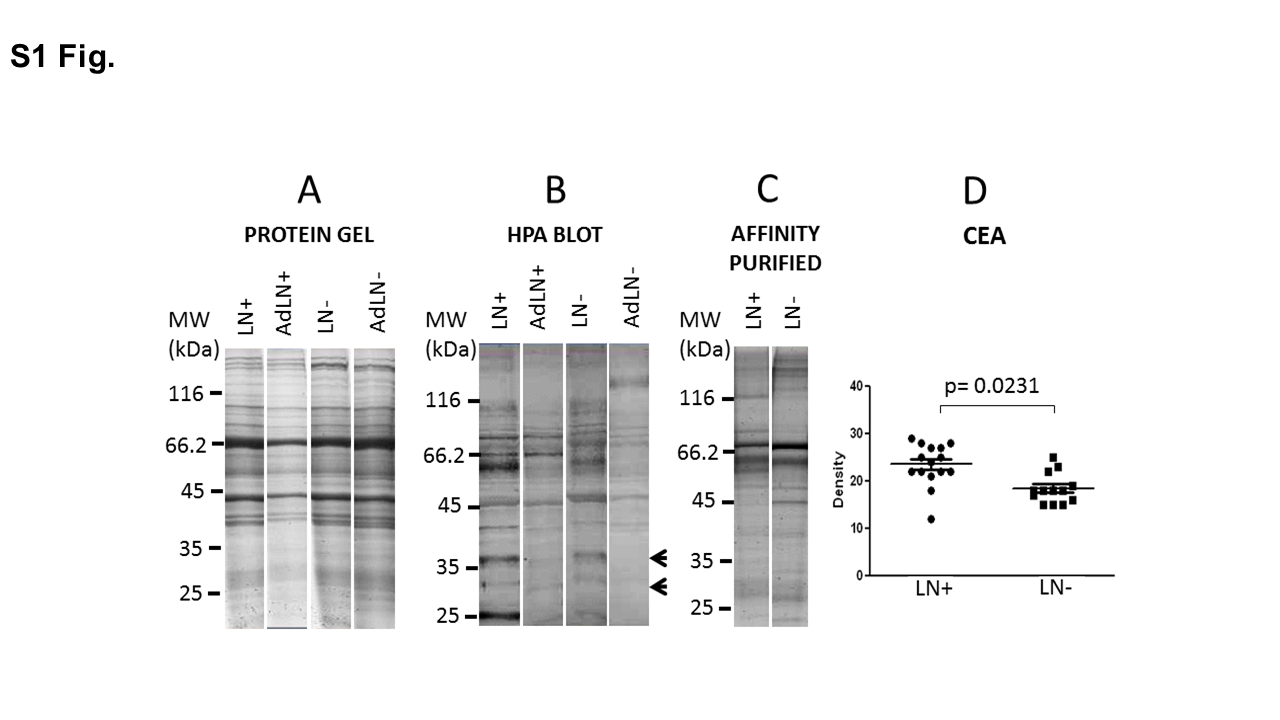

Supplement: S1 Fig — Pooled proteins were prepared from samples of patients with LN positive CRC (LN+ve), adjacent normal tissue (AdLN+ve), LN negative CRC (LN-ve) and adjacent normal tissue (AdLN-ve) and separated using 12% SDS-PAGE. Panel A: proteins, 15 μg/well, separated by SDS-PAGE and stained with colloidal Coomassie blue. Panel B: proteins separated as before, transferred to nitrocellulose and probed with 5 μg/ml biotinylated HPA, 2 μg/ml streptavidin-HRP and visualised using DAB/H2O2. The arrows indicate proteins that were present in the cancer specimens but not in adjacent normal tissue, these were subsequently identified as GATA binding protein 1 and calcium-activated chloride channel protein 1. Panel C: 10 μg of proteins purified using HPA affinity chromatography, separated by SDS-PAGE and visualised by silver staining. Panel D: Densitometric analysis of Western blots in which samples were separated by SDS-PAGE and transferred to nitrocellulose and probed with anti-CEA antibody. (TIF) [file pone.0138345.s002.TIF]
