# Supplementary material for: Identification of O-Linked Glycoproteins Binding to the Lectin Helix pomatia Agglutinin as Markers of Metastatic Colorectal Cancer
Source: PLoS One. 2015 Oct 23;10(10):e0138345. doi: 10.1371/journal.pone.0138345 (PMC4619703; doi:10.1371/journal.pone.0138345)
Supplement: S2 Fig — (DOCX) [file pone.0138345.s003.docx]

**S2 Fig.**

| **Protein Accession number** | **Protein description** | **Number of potential glycosylation &phosphorylation sites** | | | |
| --- | --- | --- | --- | --- | --- |
|  |  | **N-linked** | **O-linked** | **O-GlcNAc** | **Phosph.** |
| gi\|119571135 | GATA binding protein | 0 | 8 | 12 | 46 |
| gi\|31615935 | Fc fragment of IgA receptor | 1 | 9 | 10 | 11 |
| gi\|223468574 | Vesicle associated membrane protein | 0 | 0 | 6 | 5 |
| gi\|119590003 | Transcription factor | 0 | 5 | 5 | 26 |
| gi\|4585469 | Calcium- activated chloride channel protein 1 | 0 | 8 | 12 | 46 |
| gi\|114577910 | Annexin IV | 0 | 0 | 1 | 28 |
| gi\|157831404 | Annexin V | 0 | 0 | 4 | 28 |
